# Supplementary material for: Unveiling the Bioactive Potential of the Invasive Jellyfish Phyllorhiza punctata Through Integrative Transcriptomic and Proteomic Analyses
Source: Biomolecules. 2025 Aug 4;15(8):1121. doi: 10.3390/biom15081121 (PMC12383608; doi:10.3390/biom15081121)
Supplement: Supplementary file 1 [file biomolecules-15-01121-s001.zip › Suplementary Material/FileS3/PEPFOLD4/XP_065065930_11576/clusters.html]

 PEP-FOLD clusters


Download archive of all models  here   

|  |  |  |
| --- | --- | --- |
| # | file | sOPEP |
| # cluster 1 - Size: 16 |  |  |
|  | XP\_065065930\_11576-model1 | -14.2285 |
|  | XP\_065065930\_11576-model1.1 | -14.2285 |
|  | XP\_065065930\_11576-model1.2 | -14.2285 |
|  | XP\_065065930\_11576-model1.3 | -14.2285 |
|  | XP\_065065930\_11576-model1.4 | -14.2285 |
|  | XP\_065065930\_11576-model1.5 | -14.2285 |
|  | XP\_065065930\_11576-model1.6 | -14.2285 |
|  | XP\_065065930\_11576-model1.7 | -14.2285 |
|  | XP\_065065930\_11576-model1.8 | -14.2285 |
|  | XP\_065065930\_11576-model1.9 | -14.2285 |
|  | XP\_065065930\_11576-model1.10 | -14.2285 |
|  | XP\_065065930\_11576-model1.11 | -14.2285 |
|  | XP\_065065930\_11576-model1.12 | -14.2285 |
|  | XP\_065065930\_11576-model1.13 | -14.2285 |
|  | XP\_065065930\_11576-model1.14 | -14.2285 |
|  | XP\_065065930\_11576-model1.15 | -14.0327 |
| # cluster 2 - Size: 1 |  |  |
|  | XP\_065065930\_11576-model2 | -14.175 |
| # cluster 3 - Size: 4 |  |  |
|  | XP\_065065930\_11576-model3 | -14.1384 |
|  | XP\_065065930\_11576-model3.1 | -14.1384 |
|  | XP\_065065930\_11576-model3.2 | -14.1384 |
|  | XP\_065065930\_11576-model3.3 | -14.1384 |
| # cluster 4 - Size: 36 |  |  |
|  | XP\_065065930\_11576-model4 | -14.0766 |
|  | XP\_065065930\_11576-model4.1 | -14.0766 |
|  | XP\_065065930\_11576-model4.2 | -14.0766 |
|  | XP\_065065930\_11576-model4.3 | -14.0766 |
|  | XP\_065065930\_11576-model4.4 | -14.0766 |
|  | XP\_065065930\_11576-model4.5 | -14.0766 |
|  | XP\_065065930\_11576-model4.6 | -14.0766 |
|  | XP\_065065930\_11576-model4.7 | -14.0766 |
|  | XP\_065065930\_11576-model4.8 | -14.0766 |
|  | XP\_065065930\_11576-model4.9 | -14.0766 |
|  | XP\_065065930\_11576-model4.10 | -14.0766 |
|  | XP\_065065930\_11576-model4.11 | -14.0766 |
|  | XP\_065065930\_11576-model4.12 | -14.0766 |
|  | XP\_065065930\_11576-model4.13 | -14.0766 |
|  | XP\_065065930\_11576-model4.14 | -14.0766 |
|  | XP\_065065930\_11576-model4.15 | -14.0766 |
|  | XP\_065065930\_11576-model4.16 | -14.0766 |
|  | XP\_065065930\_11576-model4.17 | -14.0766 |
|  | XP\_065065930\_11576-model4.18 | -14.0766 |
|  | XP\_065065930\_11576-model4.19 | -14.0766 |
|  | XP\_065065930\_11576-model4.20 | -14.0766 |
|  | XP\_065065930\_11576-model4.21 | -14.0766 |
|  | XP\_065065930\_11576-model4.22 | -14.0766 |
|  | XP\_065065930\_11576-model4.23 | -14.0766 |
|  | XP\_065065930\_11576-model4.24 | -14.0766 |
|  | XP\_065065930\_11576-model4.25 | -14.0766 |
|  | XP\_065065930\_11576-model4.26 | -14.0766 |
|  | XP\_065065930\_11576-model4.27 | -14.0766 |
|  | XP\_065065930\_11576-model4.28 | -14.0766 |
|  | XP\_065065930\_11576-model4.29 | -14.0766 |
|  | XP\_065065930\_11576-model4.30 | -14.0766 |
|  | XP\_065065930\_11576-model4.31 | -14.0766 |
|  | XP\_065065930\_11576-model4.32 | -14.0766 |
|  | XP\_065065930\_11576-model4.33 | -14.0766 |
|  | XP\_065065930\_11576-model4.34 | -14.0766 |
|  | XP\_065065930\_11576-model4.35 | -14.0766 |
| # cluster 5 - Size: 43 |  |  |
|  | XP\_065065930\_11576-model5 | -14.0611 |
|  | XP\_065065930\_11576-model5.1 | -14.0611 |
|  | XP\_065065930\_11576-model5.2 | -14.0611 |
|  | XP\_065065930\_11576-model5.3 | -14.0611 |
|  | XP\_065065930\_11576-model5.4 | -14.0611 |
|  | XP\_065065930\_11576-model5.5 | -14.0611 |
|  | XP\_065065930\_11576-model5.6 | -14.0611 |
|  | XP\_065065930\_11576-model5.7 | -14.0611 |
|  | XP\_065065930\_11576-model5.8 | -14.0611 |
|  | XP\_065065930\_11576-model5.9 | -14.0611 |
|  | XP\_065065930\_11576-model5.10 | -14.0611 |
|  | XP\_065065930\_11576-model5.11 | -14.0611 |
|  | XP\_065065930\_11576-model5.12 | -14.0611 |
|  | XP\_065065930\_11576-model5.13 | -14.0611 |
|  | XP\_065065930\_11576-model5.14 | -14.0611 |
|  | XP\_065065930\_11576-model5.15 | -14.0611 |
|  | XP\_065065930\_11576-model5.16 | -14.0611 |
|  | XP\_065065930\_11576-model5.17 | -14.0611 |
|  | XP\_065065930\_11576-model5.18 | -14.0611 |
|  | XP\_065065930\_11576-model5.19 | -14.0611 |
|  | XP\_065065930\_11576-model5.20 | -14.0611 |
|  | XP\_065065930\_11576-model5.21 | -14.0611 |
|  | XP\_065065930\_11576-model5.22 | -14.0611 |
|  | XP\_065065930\_11576-model5.23 | -14.0611 |
|  | XP\_065065930\_11576-model5.24 | -14.0611 |
|  | XP\_065065930\_11576-model5.25 | -14.0611 |
|  | XP\_065065930\_11576-model5.26 | -14.0611 |
|  | XP\_065065930\_11576-model5.27 | -14.0611 |
|  | XP\_065065930\_11576-model5.28 | -14.0611 |
|  | XP\_065065930\_11576-model5.29 | -14.0611 |
|  | XP\_065065930\_11576-model5.30 | -14.0611 |
|  | XP\_065065930\_11576-model5.31 | -14.0611 |
|  | XP\_065065930\_11576-model5.32 | -14.0611 |
|  | XP\_065065930\_11576-model5.33 | -14.0611 |
|  | XP\_065065930\_11576-model5.34 | -14.0611 |
|  | XP\_065065930\_11576-model5.35 | -14.0611 |
|  | XP\_065065930\_11576-model5.36 | -14.0611 |
|  | XP\_065065930\_11576-model5.37 | -14.0611 |
|  | XP\_065065930\_11576-model5.38 | -14.0611 |
|  | XP\_065065930\_11576-model5.39 | -14.0611 |
|  | XP\_065065930\_11576-model5.40 | -14.0611 |
|  | XP\_065065930\_11576-model5.41 | -14.0611 |
|  | XP\_065065930\_11576-model5.42 | -14.0611 |
|  |  |  |
